# Combined Albumin-Bilirubin Grade and Skeletal Muscle Mass as a Predictor in Liver Cirrhosis

**DOI:** 10.3390/jcm8060782

**Published:** 2019-06-01

**Authors:** Hiroki Nishikawa, Hirayuki Enomoto, Kazunori Yoh, Yoshinori Iwata, Yoshiyuki Sakai, Kyohei Kishino, Naoto Ikeda, Tomoyuki Takashima, Nobuhiro Aizawa, Ryo Takata, Kunihiro Hasegawa, Noriko Ishii, Yukihisa Yuri, Takashi Nishimura, Hiroko Iijima, Shuhei Nishiguchi

**Affiliations:** 1 Division of Hepatobiliary and Pancreatic Disease, Department of Internal Medicine, Hyogo College of Medicine, Nishinomiya, Hyogo 663-8501, Japan; enomoto@hyo-med.ac.jp (H.E.); mm2wintwin@ybb.ne.jp (K.Y.); yo-iwata@hyo-med.ac.jp (Y.I.); sakai429@hyo-med.ac.jp (Y.S.); hcm.kyohei@gmail.com (K.K.); nikeneko@hyo-med.ac.jp (N.I.); tomo0204@yahoo.co.jp (T.T.); nobu23hiro@yahoo.co.jp (N.A.); chano_chano_rt@yahoo.co.jp (R.T.); hiro.red1230@gmail.com (K.H.); ishinori1985@yahoo.co.jp (N.I.); gyma27ijo04td@gmail.com (Y.Y.); tk-nishimura@hyo-med.ac.jp (T.N.); hiroko-i@hyo-med.ac.jp (H.I.); nishiguc@hyo-med.ac.jp (S.N.); 2 Center for clinical research and education, Hyogo College of Medicine, Nishinomiya, Hyogo 663-8501, Japan

**Keywords:** albumin-bilirubin grade, Child-Pugh classification, skeletal muscle mass, liver cirrhosis, predictability

## Abstract

We aimed to compare the prognostic impact among albumin-bilirubin (ALBI) grade, the Child-Pugh classification and our proposed combined ALBI grade and skeletal muscle mass (SMM) grading system in patients with liver cirrhosis (LC) (*n* = 468, 254 males and 214 females) using the Akaike information criterion (AIC) and time-dependent receiver operating characteristics (ROC) curve analysis. SMM was tested using bioimpedance analysis. Male subjects with skeletal muscle mass index (SMI) <7.0 cm^2^/m^2^ and female subjects with SMI <5.7 cm^2^/m^2^ were defined as having low SMM. Patients with ALBI grade 1, 2 and 3 were given 1, 2 and 3 points. Patients with and without low SMM were given 1 and 0 point, respectively. The sum of the point of ALBI (1, 2, or 3) and SMM (0 or 1) was defined as the ALBI-SMM grade. The value obtained with the AIC for survival by the ALBI-SMM grade was the lowest among three assessment methods (AIC: 513.418 in ALBI grade, 533.584 in Child-Pugh classification and 493.72 in ALBI-SMM grade). In time-dependent ROC analysis, all area under the ROCs of the ALBI-SMM grade in each time point were the highest among three assessment methods. In conclusion, the ALBI-SMM grading system can be helpful for LC patients.

## 1. Introduction

Liver cirrhosis (LC) is an end-stage status in persistent liver damage and it is often complicated by several clinical manifestations such as ascites, hepatic encephalopathy, varices due to portal hypertension or hepatocellular carcinoma (HCC), all of which can cause unfavorable clinical outcomes [1,2]. From the nutritional point of view, LC imitates starvation with an inadequate use of bodily fat and protein preservation for gluconeogenesis [2,3].

The Child-Pugh classification is commonly used worldwide for the assessment of liver functional reserve in LC patients [4]. However, the major drawback of the Child-Pugh classification is that it involves several subjective factors (ascites and hepatic encephalopathy) and interrelated factors (ascites and serum albumin) [4]. Ascites can be easily affected by diuretic use or a dehydration state. To overcome these limitations, a simple assessment method for liver functional reserve, called the albumin-bilirubin (ALBI) grade, which is calculated by only serum albumin level and total bilirubin level, has been recently proposed [5]. The predictability of ALBI grade has been verified in LC patients with or without HCC, regardless of liver disease etiologies [6,7,8,9,10,11,12]. More recently, a combined ALBI grade and other parameter grading systems has been proposed for the better prognostic ability over the ALBI grade [13,14,15,16]. 

Sarcopenia, characterized by low skeletal muscle mass (SMM) and muscle strength or physical inactivity, is currently accepted worldwide as a novel geriatric syndrome [17,18,19]. Recent studies have acknowledged the close relations between sarcopenia and adverse clinical outcomes in inflammatory diseases, malignancies, renal diseases and liver diseases [17,18,19,20,21,22,23,24,25,26,27,28,29]. Compelling evidence has shown that low SMM is a major coincidence in LC patients because of protein metabolic disorder and energy metabolic disorder (i.e., double metabolic burdens) regardless of age [17,18,23,25,30,31]. Low SMM may also have relevant implications in favoring hyperammonemia caused by advanced LC status [30,31,32]. 

Several imaging modalities including computed tomography (CT) at the L3 level, bioimpedance analysis (BIA, body composition analyzer) and dual X-ray absorptiometry can assess SMM [33,34]. BIA has particularly gained popularity because BIA is suitable for testing SMM in daily clinical practice in terms of its convenience for use, non-invasiveness, favorable cost performance, no radiation exposure and diagnostic accuracy [18,19,23,34]. However, there have been no reports examining the impact of combined ALBI grade and SMM as assessed by BIA on clinical outcomes in LC patients. In this study, we sought to compare the impact on survival among ALBI grade, the Child-Pugh classification and our proposed combined ALBI grade and SMM grading system in LC patients. 

## 2. Patients and Methods

### 2.1. Patients 

Six hundred and thirty-one LC individuals in whom BIA data were available were admitted at our institution between October 2005 and July 2018. We have routinely performed BIA testing in LC patients in whom we obtained consent for a nutritional evaluation. In this analysis, SMM was assessed by means of BIA data. Subjects with massive ascites (*n* = 27) as identified by ultrasonography or CT were excluded from this analysis because the body composition analyzer had the possibility of overestimating SMM in LC patients with a severe edematous state [30,31]. Twenty-nine patients lost prior to follow-up within 1 year after BIA were excluded from this analysis. Out of the remaining 575 subjects, 107 in whom HCC was confirmed on radiological findings at baseline or those with previous treatment history for HCC were excluded. A total of 468 subjects were therefore included in our study cohort. 

During the follow-up period after BIA, hematological and radiological tests with the aim of identifying cancer incidence or LC-related complications were periodically performed (at 3 to 6 months interval). LC was determined considering pathological data, radiologic findings and/or laboratory data [35,36,37]. When the serum albumin level showed less than 3.5 g/dL, nutritional supplementation therapies were considered [38,39]. In cases with hepatitis virus-related liver diseases, antiviral treatments such as nucleoside analogues or direct acting antivirals (DAAs) or interferon (IFN)-based treatment regimens were also considered [38]. In principal, diagnosis for HCC and strategies for HCC therapy were determined according to the current guidelines [40,41].

### 2.2. ALBI Score and ALBI Grade

The ALBI score in each subject was calculated by the following formula as reported previously [5]: ALBI score = (log 10 total bilirubin [μmol/L] × 0.66) + (serum albumin [g/L] × −0.085), while ALBI grade was classified into the following three grades: ALBI score ≤ −2.60 = grade 1, −2.60 < ALBI score ≤ −1.39 = grade 2 and ALBI score > −1.39 = grade 3 [5]. Patients with ALBI grade 1, 2 and 3 were given 1, 2 and 3 points, respectively (Table 1). 

### 2.3. Skeletal Muscle Mass and ALBI-SMM Grade

The skeletal muscle mass index (SMI) was calculated according to previous reports [19,23]. Briefly, the definition of SMI was “Sum of SMM in upper and lower extremities/(stature (m))^2^” [19,23]. Based on the recommendations of Japanese Society of Hepatology, male subjects with SMI <7.0 cm^2^/m^2^ and female subjects with SMI <5.7 cm^2^/m^2^ were defined as having low SMM [19,23]. Patients with low SMM were given 1 point and those without low SMM were given 0 point (Table 1). The sum of the point of ALBI (1, 2, or 3) and SMM (0 or 1) was defined as the ALBI-SMM grade. The ALBI-SMM grade therefore ranged from 1 to 4 (Table 1). We compared the predictive ability for survival among the ALBI grade, the Child-Pugh classification and the ALBI-SMM grade. This study protocol was acknowledged by the institutional review board in the Hyogo College of Medicine (approval no 2082) and all clinical investigations were done in compliance with the Declaration of Helsinki.

### 2.4. Statistical Analyses

Survival curves in the ALBI grade, the Child-Pugh classification and the ALBI-SMM grade were made using the Kaplan-Meier method and compared in the log-rank test. Akaike information criterion (AIC) with each assessment method was tested for comparison of survival. The fitness of the models was compared based on the AIC and the lowest value of AIC provided the best fit to the data. Furthermore, we analyzed time-dependent receiver operating characteristics (ROC) curves of ALBI grade, the Child-Pugh classification and ALBI-SMM grade for survival and compared area under the ROCs (AUCs) for these three assessment methods in each time point (1-, 2-, 3-, 4-, 5-, 6-, 7-, 8-, 9- and 10-year) [42,43,44]. Data were shown as median value (interquartile range (IQR)). The significance threshold in this analysis was *p* < 0.05 using the statistical analysis software (JMP 13 (SAS Institute Inc., Cary, NC, USA)).

## 3. Results

### 3.1. Baseline Characteristics

Demographic and clinical characteristics of the analyzed subjects (*n* = 468) were demonstrated in Table 2. The study cohort included 254 males and 214 females with the median age (IQR) of 66 (60–73) years. The median follow-up duration was 4.0 years (IQR, 2.84–7.17 years). The median (IQR) values in SMI for male and female were 7.40 (6.88–8.16) cm^2^/m^2^ and 6.0 (5.55–6.41) cm^2^/m^2^, respectively. The proportions of patients with low SMM in male and female were 35.0% (89/254) and 33.6% (72/214), respectively. Regarding Child-Pugh classification and liver disease etiologies, subjects were predominantly Child-Pugh A (341/468, 72.9%) and hepatitis C virus (HCV, 266/468, 56.8%). Small ascites was identified on radiologic findings in 59 patients (12.6%). There were 150 patients (32.1%) with ALBI grade 1, 286 patients (61.1%) with ALBI grade 2 and 32 patients (6.8%) with ALBI grade 3. While there were 104 patients (22.2%) with ALBI-SMM grade 1, 230 patients (49.1%) with ALBI-SMM grade 2, 121 patients (25.9%) with ALBI-SMM grade 3 and 13 patients (2.8%) with ALBI-SMM grade 4. In patients with ALBI grade 1, 2 and 3, 46 patients (30.7%), 102 patients (35.7%) and 13 patients (40.6%) had low SMM, respectively. The median age in patients with ALBI grade 1, 2 and 3 were 65, 67 and 67.5 years, respectively. The median age in patients with ALBI-SMM grade 1, 2, 3 and 4 were 64.5, 65, 70 and 76 years, respectively.

### 3.2. Cumulative Overall Survival Rates According to the Presence of Low SMM

**Figure 1:**
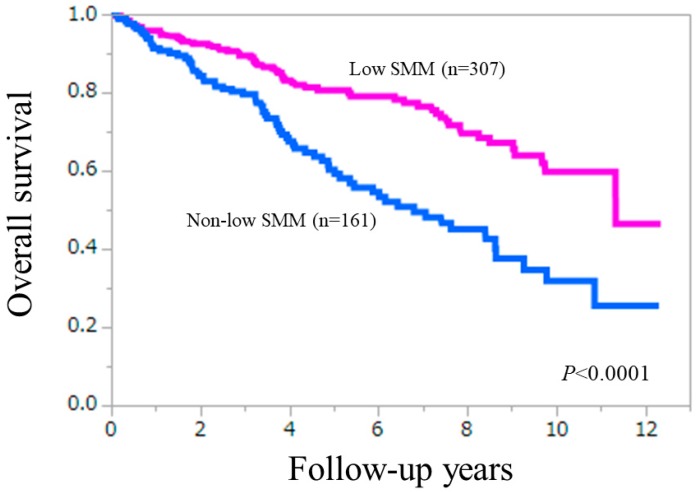
Kaplan-Meier curves in patients with low SMM (*n* = 161) and without low SMM (*n* = 307). The 3-, 5-, 7- and 10-year cumulative overall survival rates were: 79.5%, 60.3%, 49.5% and 31.8% in the low SMM group, and 89.4%, 80.6%, 76.5% and 59.7% in the non-low SMM group (*p* < 0.0001).

**Figure 2:**
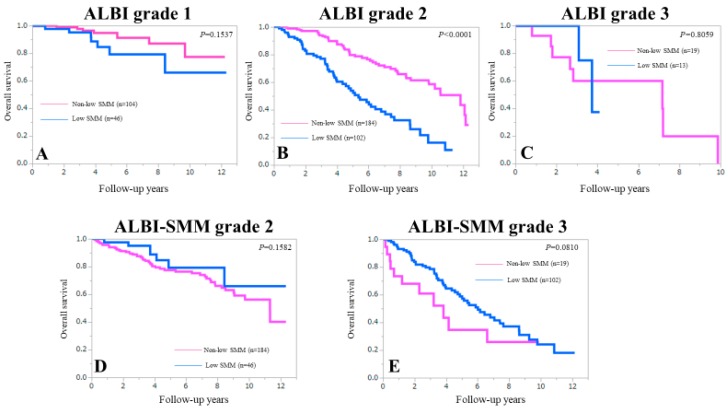
Kaplan-Meier curves in patients ALBI grade 1, 2 and 3 stratified by SMM (**A**–**C**) and Kaplan-Meier curves in patients ALBI-SMM grade 2 and 3 stratified by SMM (**D**,**E**).

Overall survival (OS) was our primary endpoint. The 3-, 5-, 7- and 10-year cumulative OS rates were 79.5%, 60.3%, 49.5% and 31.8%, respectively, in patients with low SMM and 89.4%, 80.6%, 76.5% and 59.7%, respectively, in patients without low SMM (*p* < 0.0001) (Figure 1). In patients with ALBI grade 1 and grade 3, the difference of OS between the low SMM group and the non-low SMM group did not reach significance (*p* = 0.1537 and 0.8059) (Figure 2A,C). While in patients with ALBI grade 2, the low SMM group patients had significantly lower OS rate than the non-low SMM group (*p* < 0.0001) (Figure 2B). In patients with ALBI-SMM grade 2 and 3, the difference in the two groups did not reach significance (Figure 2D,E). 

### 3.3. Causes of Death in the Low SMM Group and the Non-Low SMM Group

In the low SMM group, during the observation period, 72 patients (44.7%) died. The causes of death were 51 patients for hepatic failure, 12 for advanced HCC status and 9 for other causes. In the non-low SMM group, during the observation period, 69 patients (22.5%) died. The causes of death were 45 patients for hepatic failure, 14 for advanced HCC status and 10 for other causes.

### 3.4. Comparison of Prognostic Accuracy among Three Assessment Methods for All Cases

**Figure 3:**
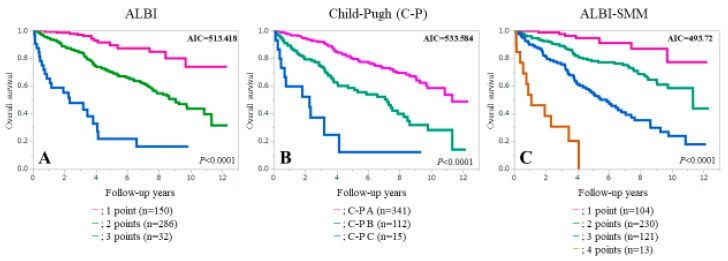
Kaplan-Meier curves according to the ALBI grade (**A**), the Child-Pugh classification (**B**) and the ALBI-SMM grade (**C**).

Patient survival was well stratified by the ALBI grade (*p* < 0.0001), the Child-Pugh classification (*p* < 0.0001) and the ALBI-SMM grade (*p* < 0.0001) for all cases (Figure 3). We compared predictive accuracy among three assessment methods (i.e., ALBI grade, the Child-Pugh classification and ALBI-SMM grade) for all cases. The AIC value for survival by ALBI-SMM grade was the lowest among three assessment methods (AIC: 513.418 in the ALBI grade, 533.584 in Child-Pugh classification and 493.72 in the ALBI-SMM grade) (Figure 3). 

### 3.5. Comparison of Prognostic Accuracy among Three Assessment Methods Stratified by Gender

**Figure 4:**
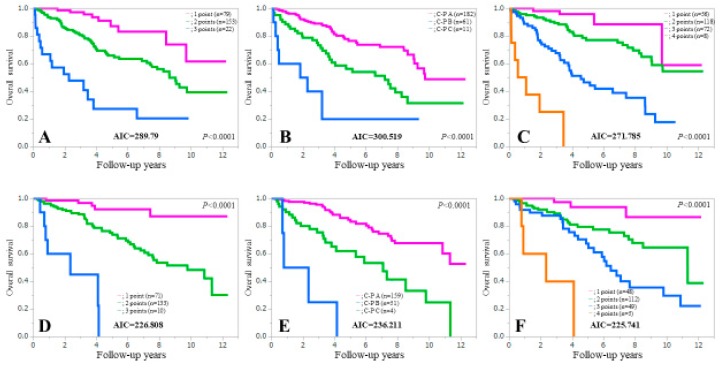
Kaplan-Meier curves in male patients (*n* = 254) according to the ALBI grade (**A**), the Child-Pugh classification (**B**) and the ALBI-SMM grade (**C**), and Kaplan-Meier curves in female patients (*n* = 214) according to ALBI grade (**D**), the Child-Pugh classification (**E**) and ALBI-SMM grade (**F**).

We also performed subgroup analyses according to gender. In male patients (*n* = 254), the AIC value for survival by the ALBI-SMM grade (AIC = 271.785) was lower than that of the Child-Pugh classification (AIC = 300.519) and the ALBI grade (AIC = 289.79) (upper part of Figure 4). Similarly, in female patients (*n* = 214), the AIC value for survival by the ALBI-SMM grade (AIC = 225.741) was lower than that of the Child-Pugh classification (AIC = 236.211) and the ALBI grade (AIC = 226.808) (lower part of Figure 4).

### 3.6. Comparison of Prognostic Accuracy among Three Assessment Methods in Patients with or without Ascites

**Figure 5:**
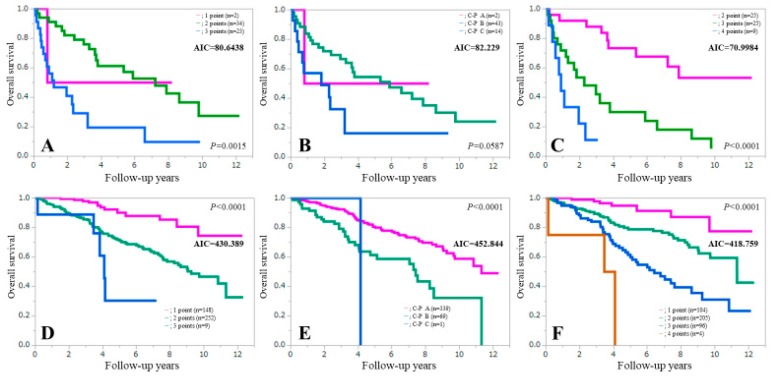
Kaplan-Meier curves in patients with ascites (*n* = 59) according to the ALBI grade (**A**), the Child-Pugh classification (**B**) and the ALBI-SMM grade (**C**), and Kaplan-Meier curves in patients without ascites (*n* = 409) according to the ALBI grade (**D**), the Child-Pugh classification (**E**) and the ALBI-SMM grade (**F**).

In patients with ascites (*n* = 59), the AIC value for survival by the ALBI-SMM grade (AIC = 70.9984) was lower than that of the Child-Pugh classification (AIC = 82.229) and the ALBI grade (AIC = 80.6438) (upper part of Figure 5). Likewise, in patients without ascites, the AIC value for survival by the ALBI-SMM grade (AIC = 418.759) was lower than that of the Child-Pugh classification (AIC = 452.844) and the ALBI grade (AIC = 430.389) (lower part of Figure 5).

### 3.7. Comparison of Prognostic Accuracy among Three Assessment Methods Stratified by Liver Disease Etiologies

**Figure 6:**
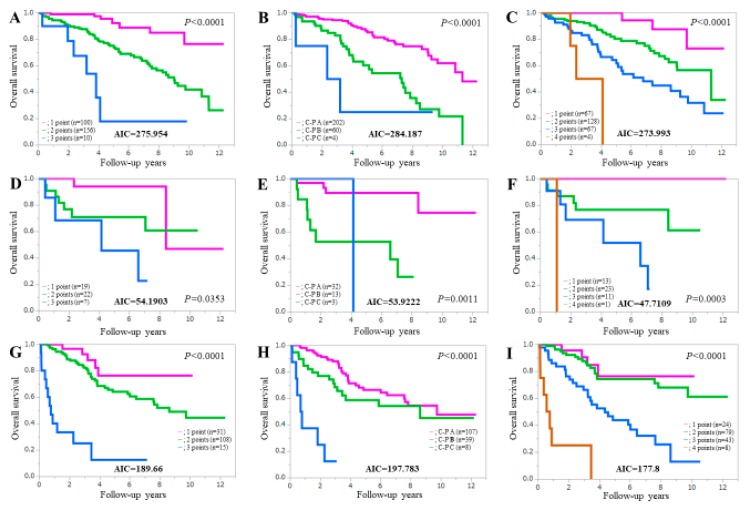
Kaplan-Meier curves in patients with HCV (*n* = 266) according to ALBI grade (**A**), the Child-Pugh classification (**B**) and ALBI-SMM grade (**C**), and Kaplan-Meier curves in patients with HBV (*n* = 48) according to the ALBI grade (**D**), the Child-Pugh classification (**E**) and the ALBI-SMM grade (**F**), and Kaplan-Meier curves in patients with other liver disease etiologies (*n* = 154) according to ALBI grade (**G**), the Child-Pugh classification (**H**) and ALBI-SMM grade (**I**).

In patients with HCV (*n* = 266), the AIC value for survival by ALBI-SMM grade (AIC = 273.993) was lower than that of the Child-Pugh classification (AIC = 284.187) and ALBI grade (AIC = 275.954) (upper part of Figure 6). Likewise, in patients with hepatitis B virus (HBV, *n* = 48), the AIC value for survival indicated by the ALBI-SMM grade (AIC = 47.7109) was lower than that of the Child-Pugh classification (AIC = 53.9222) and ALBI grade (AIC = 54.1903) (middle part of Figure 6). In patients with other liver disease etiologies (*n* = 154), the AIC value for survival indicated by the ALBI-SMM grade (AIC = 177.8) was lower than that of the Child-Pugh classification (AIC = 197.783) and the ALBI grade (AIC = 189.66) (lower part of Figure 6).

In patients with HCV, 155 patients (58.3%) achieved a sustained virological response (SVR) during the follow-up period: 44 patients received IFN-based therapies and 111 patients received DAA-based therapies.

### 3.8. Comparison of Prognostic Accuracy among Three Assessment Methods According to Age

**Figure 7:**
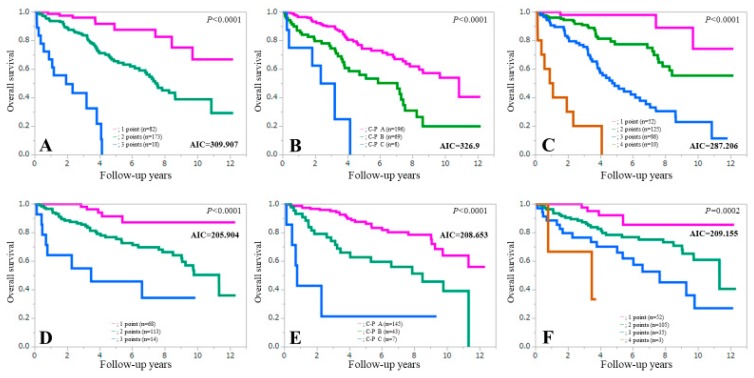
Kaplan-Meier curves in patients aged 65 years or more (*n* = 273) according to the ALBI grade (**A**), the Child-Pugh classification (**B**) and the ALBI-SMM grade (**C**), and Kaplan-Meier curves in patients aged less than 65 years (*n* = 195) according to the ALBI grade (**D**), the Child-Pugh classification (**E**) and the ALBI-SMM grade (**F**).

In patients aged 65 years or more (elderly persons, *n* = 273), the AIC value for survival by ALBI-SMM grade (AIC = 287.206) was lower than that of the Child-Pugh classification (AIC = 326.9) and ALBI grade (AIC = 309.907) (upper part of Figure 7). While in patients aged less than 65 years (*n* = 195), the AIC value for survival indicated by the ALBI grade (AIC = 205.904) was lower than that of the Child-Pugh classification (AIC = 208.653) and the ALBI-SMM grade (AIC = 209.155) (lower part of Figure 7).

### 3.9. Comparison of Prognostic Accuracy among Three Assessment Methods Using Time-Dependent ROC Analysis

**Figure 8:**
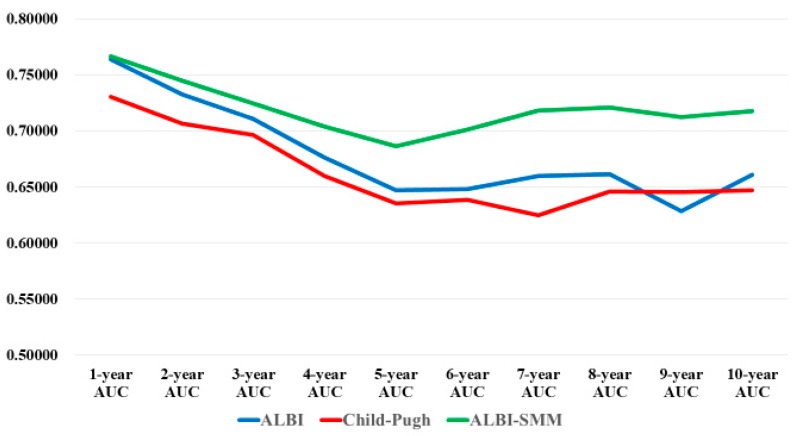
Area under the receiver operating characteristic curves (AUCs) in time-dependent ROC analysis in the ALBI grade, the Child-Pugh classification and the ALBI-SMM grade in each time point.

Results for time-dependent ROC analyses at 1-, 2-, 3-, 4-, 5-, 6-, 7-, 8-, 9- and 10 year of the ALBI grade, the Child-Pugh classification and the ALBI-SMM grade for all cases were shown in Table 3 and Figure 8. All AUCs of the ALBI-SMM grade in each time point were higher than those of the Child-Pugh classification and the ALBI grade, indicating that the ALBI-SMM grade had superior predictive ability for survival over the Child-Pugh classification and the ALBI grade.

## 4. Discussion

Traditionally, the Child-Pugh scoring system has been used to evaluate the liver functional reserve. However, assessment of the severity of ascites and hepatic encephalopathy may be subjective and difficult to be consistently scored by different evaluators. Since the introduction of the ALBI grade, which is a simple objective assessment method, numerous studies have confirmed the better accuracy of the ALBI grade over the Child-Pugh classification in predicting liver functional reserve as well as in predicting clinical outcomes [6,7,8,9,10,11,12]. On the other hand, low SMM is currently accepted as an adverse predictor in LC patients [17,18,19,20,21,22,23,24,25,26,27,28,29]. As described above, predictive models combining the ALBI grade and other parameters such as PALBI (combined ALBI and platelet count) or ALBI-T (combined ALBI and tumor status) have been put forward [13,14,15,16]. The ALBI grade does not incorporate SMM and we hypothesized that combination of these have potential to exert more accurate predictability for LC patients. In this study, we demonstrated the superior predictive accuracy of our proposed ALBI-SMM grading system over the Child-Pugh classification and the ALBI grade not only by comparing AICs in three assessment methods but also using ROC analysis with a consideration of time dependence. The major strength of this study is its large sample size (*n* = 468). 

Time-dependent ROC analysis appears to be useful for estimating the predictability of markers [44]. Tada, et al. demonstrated that HBV core-related antigen was an excellent predictor of HCC development for chronic hepatitis B patients without nucleoside analogue therapy using time-dependent ROC analysis [43]. In the conventional ROC curve analysis, the patient’s clinical outcome (i.e., alive or dead) is defined once a marker value is tested and it is assumed to be fixed during the entire study period. Most studies for survival analysis involve a long-time observation period and the status of an individual (alive or dead) is updated at every time points in time-dependent ROC curve analysis [44]. An AUC can be calculated at each time point and the marker’s predictability can be compared. In our analysis, AUCs in all time points of the ALBI-SMM grade was consistently higher than those of the ALBI grade or the Child-Pugh classification, suggesting the excellent predictability of our proposed grading system. 

With regard to the comparison of predictability between the ALBI grade and the Child-Pugh classification, all AICs in the ALBI grade were lower than those in the Child-Pugh classification except for patients with HBV, while time dependent ROC analysis showed the almost consistent higher AUCs of the ALBI grade compared with those of the Child-Pugh classification. Our current results were in agreement with previous reports [5,6,7,8,9,10,11,12]. 

Recently, the advent of oral DAA agents has dramatically improved SVR rates for HCV therapy, providing SVR rates over 95% with shorter treatment duration of HCV therapy and a favorable safety profile [45,46]. In this study, 155 LC patients (58.3%) achieved SVR during the follow-up period. Increased serum albumin level and SMM can be observed in HCV patients with SVR [47]. Nevertheless, the ALBI-SMM grade had the lowest AIC among three assessment methods in our HCV patients, suggesting the robustness of our proposed grading system. 

One weak point of our grading system is that its impact was diminished in patients aged less than 65 years as shown in Figure 7D–F. These results may be attributed to the difference of incidence of low SMM in patients 65 years or more (43.2%, 118/273) and less than 65 years (22.1%, 43/195). The incidence of low SMM can increase with aging [23]. Our proposed grading system may not be useful in younger LC subjects. 

Several limitations are inherent in the present analysis. First, this is a retrospective and single center observational study and the usefulness of our proposed ALBI-SMM grading system should be confirmed in other independent cohorts. Secondly, due to the limitation for evaluating SMM in BIA, subjects with severe ascites who are highly suspected to be involved in low SMM were excluded from our study cohort. Consequently, the number of our Child-Pugh C patients was pretty small compared with that of Child-Pugh A or B patients, leading to bias. Thirdly, our study subjects were limited to LC patients without HCC; whether our proposed grading system could be extrapolated to HCC patients or non-LC patients requires additional research. However, our study results denoted that ALBI-SMM will be a helpful grading system, at least in elderly LC patients. 

In conclusion, we identified the ALBI-SMM grade as the strongest with ability to separate LC patients into different prognostic groups. Our proposed ALBI-SMM grading system appears to be helpful for estimating prognosis in LC patients. 

## Figures and Tables

**Table 1:** Our proposed combined ALBI grade and skeletal muscle mass grading system.

	Points			
ALBI grade	1	2	3	
Skeletal muscle mass (SMM)	0	1		
ALBI-SMM grade	1	2	3	4

**Table 2:** Baseline data (*n* = 468).

Variables	Number or Median (Interquartile Range)
Age (years)	66 (60, 73)
Gender, male/female	254/214
Body mass index (kg/m^2^)	23.1 (20.5, 25.8)
Skeletal muscle mass index (cm^2^/m^2^), male	7.4 (6.9, 8.16)
Skeletal muscle mass index (cm^2^/m^2^), female	6.0 (5.55, 6.41)
Causes of liver disease	48/266/154
Hepatitis B/Hepatitis C/others	
Child-Pugh classification, A/B/C	341/112/15
ALBI grade, 1/2/3	150/286/32
Total bilirubin (mg/dL)	1.0 (0.7, 1.4)
Serum albumin (g/dL)	3.7 (3.2, 4.1)
Prothrombin time (PT, %)	77.3 (66.65, 86.95)
PT-international normalized ratio (INR)	1.16 (1.08, 1.26)
Platelets (×10^4^/mm^3^)	9.9 (7.1, 14.2)
Serum creatinine (mg/dL)	0.67 (0.57, 0.79)
Serum sodium (mmol/L)	140 (138, 141)
Total cholesterol (mg/dL)	152 (130, 177)
Triglyceride (mg/dL)	83 (62, 111)
AST (IU/L)	38 (27, 58)
ALT (IU/L)	30 (20, 49)
Fasting blood glucose (mg/dL)	102 (93, 119)
Branched-chain amino acid to tyrosine ratio	4.17 (3.265–5.445)
Ascites, yes/no	59/409

ALBI; albumin-bilirubin, AST; aspartate aminotransferase, ALT; alanine aminotransferase.

**Table 3:** Area under the receiver operating characteristic curve (AUC) for each time point in the ALBI grade, the Child-Pugh classification and the ALBI-SMM grade.

	**1-Year AUC**	**2-Year AUC**	**3-Year AUC**	**4-Year AUC**	**5-Year AUC**
ALBI grade	0.76386	0.73252	0.71098	0.67643	0.64719
Child-Pugh classification	0.73065	0.70642	0.69663	0.66004	0.63568
ALBI-SMM grade	0.76641	0.74486	0.72440	0.70407	0.68639
	**6-Year AUC**	**7-Year AUC**	**8-Year AUC**	**9-Year AUC**	**10-Year AUC**
ALBI grade	0.64814	0.65992	0.66159	0.62852	0.66108
Child-Pugh classification	0.63889	0.62503	0.64606	0.64540	0.64710
ALBI-SMM grade	0.70133	0.71813	0.72086	0.71265	0.71749

## Abbreviations

LC; liver cirrhosis, HCC; hepatocellular carcinoma, ALBI; albumin-bilirubin, SMM; skeletal muscle mass, CT; computed tomography, BIA; bioimpedance analysis, DAA; direct acting antiviral, IFN; interferon, SMI; skeletal muscle mass index, AIC; Akaike information criterion, ROC; receiver operating characteristics, AUC; area under the ROC, IQR; interquartile range, HCV; hepatitis C virus, HBV; hepatitis B virus, OS; overall survival, SVR; sustained virological response
